# Identification of a Broadly Cross-Reactive Epitope in the Inner Shell of the Norovirus Capsid

**DOI:** 10.1371/journal.pone.0067592

**Published:** 2013-06-21

**Authors:** Gabriel I. Parra, JoLynn Azure, Renate Fischer, Karin Bok, Carlos Sandoval-Jaime, Stanislav V. Sosnovtsev, Peter Sander, Kim Y. Green

**Affiliations:** 1 Caliciviruses Section, Laboratory of Infectious Diseases, National Institute of Allergy and Infectious Diseases, National Institutes of Health, DHHS, Bethesda, Maryland, United States of America; 2 Clinical Diagnostics, R-Biopharm AG, Darmstadt, Germany; University of North Carolina School of Medicine, United States of America

## Abstract

Noroviruses are major pathogens associated with acute gastroenteritis. They are diverse viruses, with at least six genogroups (GI-GVI) and multiple genotypes defined by differences in the major capsid protein, VP1. This diversity has challenged the development of broadly cross-reactive vaccines as well as efficient detection methods. Here, we report the characterization of a broadly cross-reactive monoclonal antibody (MAb) raised against the capsid protein of a GII.3 norovirus strain. The MAb reacted with VLPs and denatured VP1 protein from GI, GII, GIV and GV noroviruses, and mapped to a linear epitope located in the inner shell domain. An alignment of all available VP1 sequences showed that the putative epitope (residues 52–56) is highly conserved across the genus *Norovirus*. This broadly cross-reactive MAb thus constitutes a valuable reagent for the diagnosis and study of these diverse viruses.

## Introduction

Noroviruses are the major cause of non-bacterial epidemic gastroenteritis and an important cause of endemic gastroenteritis. Recent estimates have shown that noroviruses could be responsible for up to 200,000 deaths each year in children under 5 years of age in developing countries [1], [2].

Noroviruses are non-enveloped viruses that possess a single-stranded, positive-sense RNA genome that is divided into three open reading frames (ORF). ORF1 encodes the nonstructural proteins, ORF2 encodes the major capsid protein (VP1) and ORF3 encodes a small basic protein (VP2), which has been associated with capsid stability [2], [3]. The expression of VP1 results in the self-assembly of virus-like particles (VLPs) that have been shown to be morphologically and antigenically similar to the native virion [4], [5]. Recent clinical studies have shown that norovirus VLPs are safe, induce humoral and mucosal immune responses, and prevent illness in adults challenged orally with the same strain [6]–[8].

The norovirus genome is enclosed within an icosahedral capsid, which is formed by 180 molecules of VP1 organized into 90 dimers. Each VP1 monomer is divided into two major structural domains: the shell (S) and protruding (P). The S domain forms the icosahedral scaffold from which the P domain projects to form arch-like structures [9]. Because of the exposed nature of the P domain, it contains the determinants of cell attachment as well as major antigenic sites [10]–[14].

Based on amino acid differences of the VP1, noroviruses are currently classified into six different genogroups (GI-GVI), which present over 30 genotypes [2], [15]. Thus, like many other RNA viruses [16], noroviruses exhibit marked diversity that has hampered the development of a broadly cross-reactive vaccine as well as a universal method of detection. Human noroviruses have not yet been propagated in cell culture. Norovirus VLPs have facilitated the development of hyperimmune sera and monoclonal antibodies (MAbs), which have been useful for characterization of antigenic sites of the virus capsid and for the development of detection systems [10], [11], [14], [17]–[26]. Immunoassays for rapid and easy detection of norovirus have been improved with the inclusion of new broadly cross-reactive antibodies [23].

Here we describe the identification of a novel broadly cross-reactive MAb that targets five residues (52–56) of the S domain that are conserved among all strains from the six genogroups described for noroviruses. This broadly cross-reactive MAb constitutes a valuable reagent for diagnosis and study of human and animal noroviruses.

## Materials and Methods

### Ethics Statement

Animal experiments and MAb production were carried out at BioGenes GmbH (Berlin, Germany), following German and European guidelines as well as the NIH/OLAW Animal Welfare assurance guidelines (#A5755-01). Animal protocols were approved by the Institutional Animal Care and Use Committee of BioGenes GmbH. All efforts were made to reduce stress and minimize suffering.

### Expression and purification of VLPs

The expression and purification of the VLPs from Hu/NoV/GI.1/Norwalk/1968/US (NV), Hu/NoV/GI.3/DesertShield395/1990/US (DSV), Hu/NoV/GII.1/Hawaii/1971/US (HV), Hu/NoV/GII.2/SnowMountain/1976/US (SMV), Hu/NoV/GII.3/Toronto24/1991/CA (TV), Hu/NoV/GII.4/MD2004-3/2004/US (MD2004-3), and Hu/NoV/GIV.1/SaintCloud624/1998/US (SCV), and Mink Vesivirus are described elsewhere [4], [5], [14], [27], [28]. A recombinant plasmid encoding the VP1 protein of MNV-1 was used to transform *Escherichia coli* (strain BL21), and was grown in kanamycin until it reached an OD of 0.6. Then isopropylthio-galactoside (Invitrogen) was added to a final concentration of 0.8 mM to induce protein expression overnight at 37 °C. To purify recombinant MNV-1 VP1 protein, immobilized metal affinity chromatography was performed, following the protocol described by Holzinger et al. [29].

### Production of MAb

BALB/c mice were immunized intraperitoneally with TV VLPs in equal volumes of Freund's complete adjuvant (priming) or incomplete adjuvant (boosting). After testing the serum titers, mice were sacrificed by inhalational isoflurane anesthesia followed by cervical dislocation, and spleen cells were isolated and fused with myeloma cells as described previously [30]. All positive clones producing IgG against TV VLPs were selected for further cloning by limiting dilution. The reactivity of the single clone hybridoma supernatants was tested against TV VLPs and positive-cells were collected for further characterization.

### Enzyme-linked immunosorbent assay (ELISA)

The reactivity of a MAb (designated TV20) with norovirus VLPs was examined by ELISA as described elsewhere [14]. Briefly, 96-well polyvinyl microtiter plates (Thermo, Milford, MA) were coated with 50 ng/well of purified VLPs and incubated overnight at 4 °C. Wells incubated with PBS alone were used as a negative control for MAb binding. Wells were washed with PBS containing 0.1% Tween 20 (PBS-T) and blocked with PBS 5% fat free milk for 1 h at room temperature (RT). MAb TV20 was used at 5 µg/mL and adsorbed for 2 h at RT, and detected with horseradish peroxidase (HRP)–conjugated goat anti-mouse immunoglobulin G (1∶2,000 dilution; KPL, Gaithersburg, MD) and 2,2′-azino-bis(3-ethylbenzthiazoline-6-sulphonic acid) (ABTS) (KPL). The binding of VLPs to the plate was confirmed with guinea pig hyperimmune sera (1∶500 dilution) raised against each of the homologous VLPs; except for GII.2 VLPs in which GII.1 hyperimmune serum was used.

### Western blot analyses

Reactivity of MAb TV20 with Norovirus VLPs was analyzed by Western blot. Briefly, 2.5 mg of VLPs were mixed with Novex® 2× Tris-Glycine SDS loading buffer (Invitrogen, Carlsbad, CA), boiled for 5 min at 95 °C, and separated by SDS-PAGE. The proteins were electroblotted onto a nitrocellulose membrane using the iBlot® Dry Blotting System (Invitrogen). The membrane was blocked with PBS 5% fat free milk for 1 h at RT. MAb TV20 (1∶10,000) was adsorbed for 2 h at RT and the binding was detected with HRP–conjugated goat anti-mouse immunoglobulin G (1∶2,000) and SuperSignal® West Pico Chemiluminescent Substrate (Thermo Scientific, Rockford, IL).

### Peptide screening

Libraries of 17-mer overlapping biotinylated-peptides (offset of 5 residues) from the Shell domain of the NV major capsid protein were used to characterize the binding sites of the MAb as recommended by the manufacturer (Mimotopes, Melbourne, Australia). Briefly, biotinylated-peptides were incubated overnight at 4°C in NeutriAvidin-coated plates (Thermo Scientific), and the excess was washed with PBS-T 1% bovine serum albumin (BSA; Sigma, MO). MAb TV20 was incubated for 2 h in PBS-T BSA 0.1% and reactivity was determined by incubation with a HRP-conjugated goat anti-mouse immunoglobulin G (1∶2,000 dilution; KPL), and peroxidase substrate ABTS (KPL). Biotinylated Norwalk VLPs were used for binding (positive) control, and non-biotinylated Norwalk VLPs as negative control.

### Cloning of putative epitope

The putative epitope of MAb TV20 was inserted into the C-terminal end of the green fluorescent protein (GFP) with primers engineered to carry the MAb TV20 epitope and specific restriction sites (Table 1). A pCI-GFP was used as a template and amplicons were digested with XbaI and SalI and cloned into a pCI vector with Rapid DNA Ligation Kit (Roche Applied Science, Germany). Each of the products was transformed into TOP10 competent cells (Invitrogen). Transformed cells were grown overnight in LB plates with carbenicillin (50 µg/mL), and individual colonies were used for plasmid amplification and purification. The resulting plasmids were subjected to sequencing analysis to verify the presence of the MAb TV20 epitope.

**Table 1:** Primers used for cloning and site-directed mutagenesis*

Primer Name	Sequence 5′ → 3′
TV20(51–57)f	GGATCACACATGGCATGGACGAGCTGTACAAGCCTATTGATCCCTGGATAATTTGAGCGGCCGCCGACTAGTGAGCTCGTCGACCC
TV20(51–57)r	GGGTCGACGAGCTCACTAGTCGGCGGCCGCTCAAATTATCCAGGGATCAATAGGCTTGTACAGCTCGTCCATGCCATGTGTGATCC
TV20(52–56)f	GGATCACACATGGCATGGACGAGCTGTACAAGATTGATCCCTGGATATGAGCGGCCGCCGACTAGTGAGCTCGTCGACCC
TV20(52–56)r	GGGTCGACGAGCTCACTAGTCGGCGGCCGCTCATATCCAGGGATCAATCTTGTACAGCTCGTCCATGCCATGTGTGATCC
TV20(52A)f	GACAAGTTAATCCT**GC**TGATCCCTGGATAAT
TV20(52A)r	ATTATCCAGGGATCA**GC**AGGATTAACTTGTC
TV20(53A)f	GACAAGTTAATCCTATTG**C**TCCCTGGATAAT
TV20(53A)r	ATTATCCAGGGA**G**CAATAGGATTAACTTGTC
TV20(54A)f	CAAGTTAATCCTATTGAT**G**CCTGGATAATTA
TV20(54A)r	TAATTATCCAGG**C**ATCAATAGGATTAACTTG
TV20(55A)f	GTTAATCCTATTGATCCC**GC**GATAATTAATA
TV20(55A)r	TATTAATTATC**GC**GGGATCAATAGGATTAAC
TV20(56A)f	CCTATTGATCCCTGG**GC**AATTAATAATTTTGTG
TV20(56A)r	CACAAAATTATTAATT**GC**CCAGGGATCAATAGG

* Insertions of the MAb TV20 epitope in pCI-GFP vector are underlined. Point mutations in pCI-NV-VP1 are shown in bold.

### Mutagenesis analyses

The VP1 coding region (ORF2) of the NV was amplified and cloned into a pCI vector using the restriction sites *Sal*I and *Not*I as described elsewhere [14]. Site-directed mutagenesis of pCI-NV was performed using the QuikChange Site-Directed Mutagenesis Kit (Stratagene, La Jolla, CA), and complementary forward and reverse primers that carried the nucleotide mutations (Table 1). The restriction enzyme *Dpn*I (10 U/ µl) was used to digest the parental DNA. Each of the mutated products was transformed into XL10-Gold® ultracompetent cells (Stratagene). Transformed cells were grown overnight in LB plates with carbenicillin (50 µg/ml), and individual colonies were used for plasmid amplification. The resulting plasmids were subjected to sequencing analysis to verify the entire VP1 coding region and confirm the presence of introduced mutations.

### Immunofluorescence microscopy

Vero cells were plated in 96-well plates at 80,000 cells/well, and infected with modified vaccinia virus expressing bacteriophage T7 RNA polymerase (MVA-T7) at MOI = 1 PFU/cell for 1–2 h [31]. After infection, cells were transfected with 400 ng/well of each DNA construct, Lipofectamine™ LTX and Plus Reagent (Invitrogen) following manufacturer's recommendations. Cells were incubated for 24 h and fixed with cold methanol for 20 min. The optimal dilution (1∶200) of MAb was determined by serial dilutions. Goat anti-mouse immunoglobulin G (H+L) conjugated with Alexa Fluor 594 (Molecular Probes-Invitrogen, Carlsbad, CA) was used for detection. Expression of GFP (prior to fixation) or guinea pig hyperimmune sera raised against Norwalk VLPs and goat anti-guinea pig immunoglobulin G (H+L) conjugated with Alexa Fluor 594 (Molecular Probes-Invitrogen) were used to confirm the expression of the vectors. RAW264.7 cells infected with murine norovirus (MNV-1; MOI = 1), were used to assess the reactivity with MAb TV20 following the same procedure as described above.

### Blocking of VLPs binding to synthetic ABH histo-blood group antigens (HBGA) by MAb

The ability of MAb TV20 to block the binding of MD2004-3 to B carbohydrate was determined as described previously [14]. First, different concentrations of MAb (10-fold dilutions, starting at 1.5 µg/ml) were pre-incubated with 1.5 µg/ml of MD2004-3 VLPs for 2 h. The VLPs (in the presence or absence of MAb) were added to carbohydrate coated-plates and incubated for 1 h. The binding of captured MD2004-3 VLPs was determined by incubation with guinea pig hyperimmune serum (1∶2,000 dilution), followed by incubation with a HRP-conjugated goat anti-guinea pig immunoglobulin G (1∶2,000 dilution; KPL), and peroxidase substrate ABTS (KPL). The percentage of blocking was calculated using the value obtained from VLPs that were not pre-incubated with MAb. The blocking of ≤50% of binding was considered the cut-off value [32], [33].

### Protein modeling

The solved structure of the VP1 of NV (GI.1) (Protein Data Bank [PDB] accession number (1IHM) was used to identify the residues involved in the binding with MAb TV20 and visualized by using UCSF Chimera [34].

## Results and Discussion

**Figure 1:**
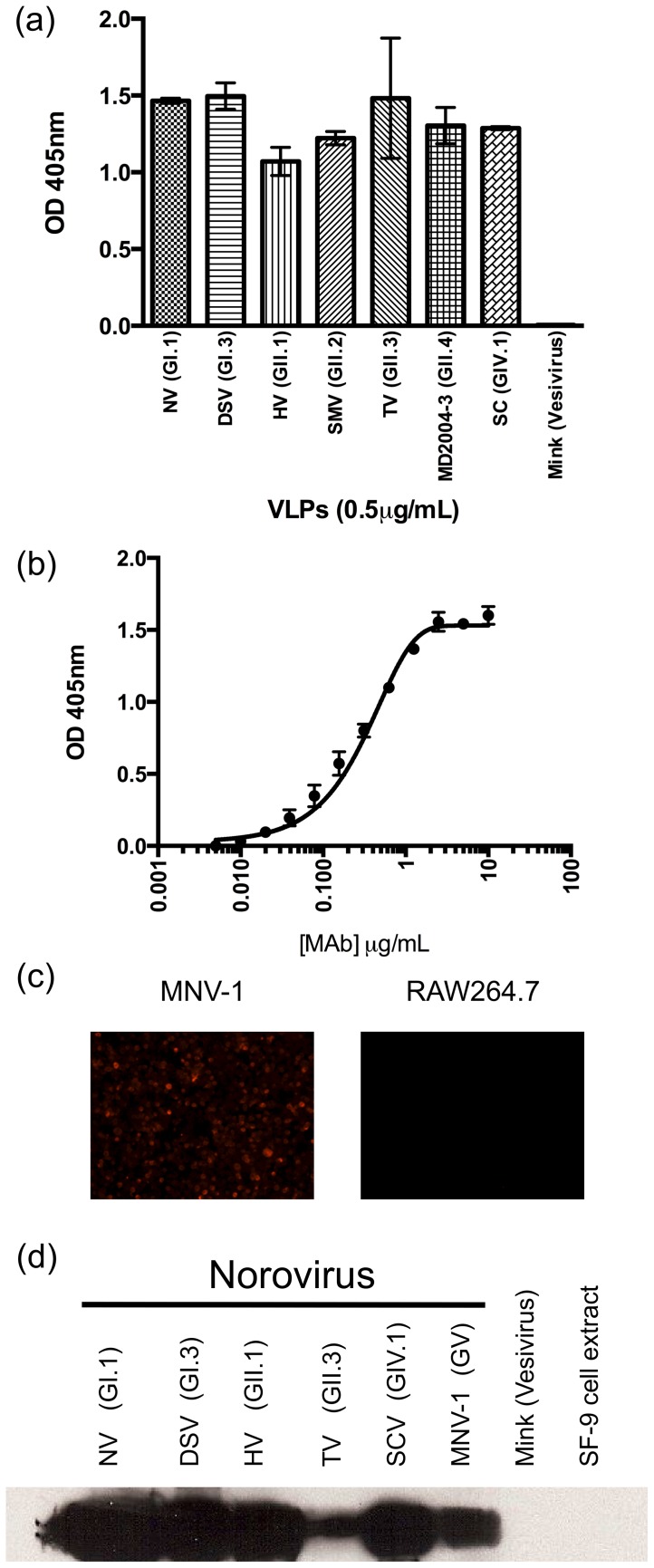
Monoclonal antibody (MAb) TV20 cross-reacts with noroviruses from different genogroups. (A) Reactivity of MAb TV20 against norovirus VLPs representing three genogroups (GI, GII, and GIV) as determined by ELISA. Mink calicivirus VLPs were used as a negative control. (B) Representative concentration curve of the MAB TV20 and NV VLPs. (C) Reactivity of MAb TV20 against RAW264.7 cells infected with MNV-1. Uninfected cells (right panel) were used as negative control. (C) Western blot of MAb TV20 against representative norovirus VP1s (GI, GII, GIV, and GV). VLPs nomenclature and experimental details are described in *Materials and Methods*.

After immunization with VLPs from the human strain Hu/GII.3/Toronto24/1991/CA, a murine MAb (TV20) was identified that reacted with norovirus VLPs from strains representing the three genogroups that infect humans (i.e. GI, GII and GIV; Fig. 1a). Titration curve shows that 40 ng/mL of the MAb can still detected VLPs by ELISA (Fig. 1b), being the EC50 = 0.3048 µg/mL. Interestingly, MNV-1 infecting RAW264.7 cells was also detected by immunofluorescence using MAb TV20 (Fig. 1b).

**Figure 2:**
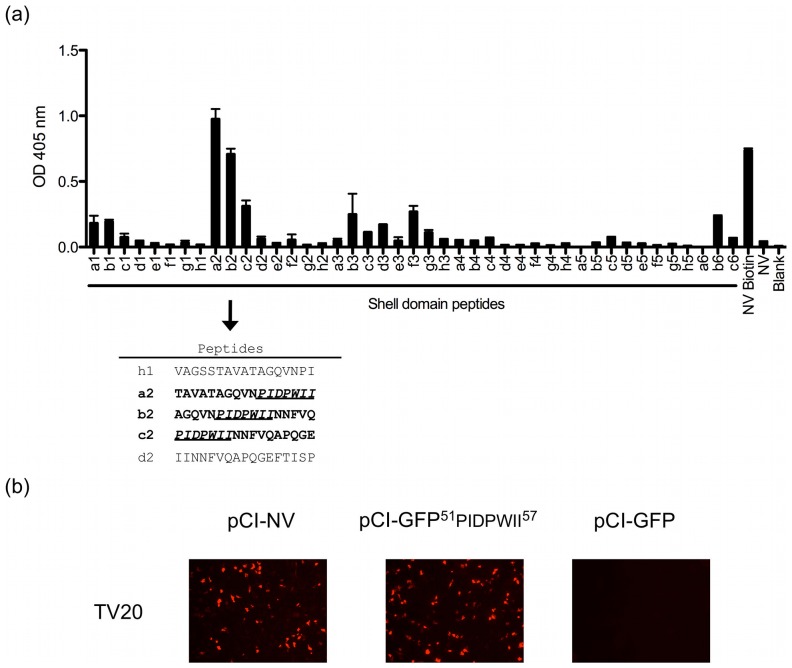
MAb TV20 binds to the Shell domain of the capsid protein. (A) Reactivity of MAb TV20 with library of peptides corresponding to the Shell domain of NV. Positive peptides span residues 41–67 from NV VP1 protein. The common residues of the three positive peptides are underlined. (B) Reactivity of MAb TV20 with cells expressing the putative epitope (residues 51–57) fused at the C-terminus of the green fluorescent protein. Experiments were performed as described in *Materials and Methods*.

Immunoblots performed with the denatured VP1 protein from different norovirus strains suggested that the MAb TV20 binds to a linear epitope conserved among the tested strains (Fig. 1c). Therefore, because of the cross-reactive nature of the MAb and the known sequence conservation in the S domain [9], we tested by ELISA the reactivity of the MAb TV20 against a library of overlapping linear peptides (17-mer length) that span the entire S domain of the NV VP1. We found that two consecutive peptides reached optical density (OD) values similar to the positive control, while a third adjacent peptide showed OD values >7 times higher than the negative control (Fig. 2a). The three consecutive peptides (a2-c2) that yielded a positive value spanned residues 41–67 of the VP1 protein from NV. A detailed analysis of the sequence of these three peptides showed that they share seven common residues (^51^PIDPWII^57^; Fig. 2a). To confirm the data obtained with the peptide library, we incorporated the nucleotide sequence encoding the putative epitope (aa 51–57) at the 3′ end of a GFP gene and cloned the engineered sequences into a eukaryotic expression vector. GFP expression (prior to methanol cell monolayer fixation) was used to confirm the expression of the vectors. Cells that transiently expressed the peptide (aa 51–57) fused with GFP were positive for reactivity with MAb TV20 by immunofluorescence (Fig. 2b), confirming the peptide scanning data and showing also that the epitope could be transferred into an expression system.

**Figure 3:**
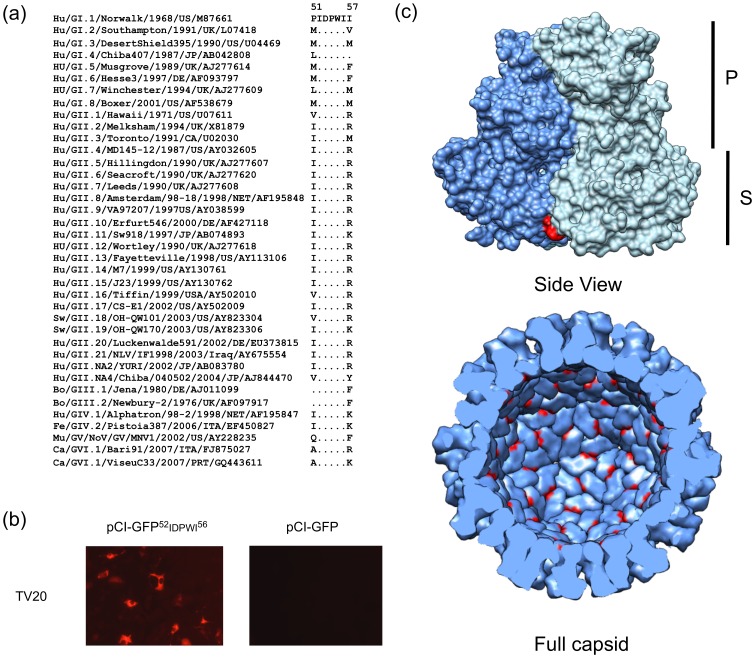
Putative epitope from MAb TV20 is highly conserved among noroviruses. (A) Amino acid alignment (residues 51–57) from representative noroviruses from the six genogroups described. Same residue is represented by a dot. MAb TV20 reactivity is indicated on the right-hand side: “+” positive, “NT” not tested. (B) Reactivity of MAb TV20 with cells expressing the putative epitope (residues 52–56) fused at the C-terminus of the green fluorescent protein. Experiments were performed as described in *Materials and Methods*. (C) Structure of NV VP1 (PDB: 1IHM) showing the location of the putative epitope from MAb TV20 (highlighted in red) in the context of the VP1 dimer and the whole capsid. The domains of the VP1 are shown on the right-hand side.

**Figure 4:**
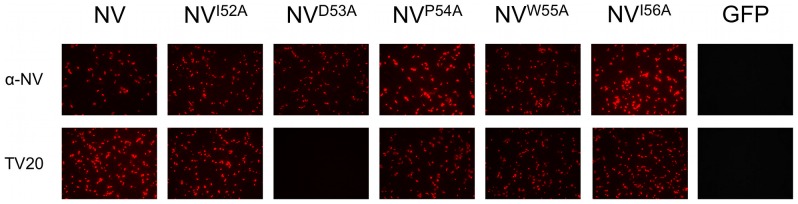
Reactivity of MAb TV20 with NV capsid proteins carrying point mutations. Immunofluorescence staining results from Vero cells transfected with different DNA constructs. Mutations of the VP1 from NV (pCI-NV) were introduced by using specific primers and the QuikChange Site-Directed Mutagenesis Kit (Stratagene, La Jolla, CA) following the manufacturer's recommendations. pCI expressing GFP was used as negative control. Hyperimmune sera against NV (α-NV) was used as a control to detect VP1 expression.

An alignment of the VP1 from representative strains of all genotypes described thus far for the genus *Norovirus* showed that multiple substitutions were found at residues 51 and 57 (Fig. 3a), while residues 52–56 were highly conserved. The cross-reactive nature of MAb TV20 suggested that the conserved residues might be required for binding. This was confirmed by cloning and transient expression of GFP fused to the peptide ^52^IDPWI^56^ at its C-terminal end (Fig. 3b). Analysis of the X-ray structure of NV VP1 protein revealed that residues 52–56 are located in an exposed area at the beginning of the S domain, but inside the viral particle (Fig. 3c). In order to determine which residues are required for MAb TV20 binding, we performed alanine scanning mutagenesis of residues 52–56 of the NV VP1. Only construct D53A, in which the aspartic acid at position 53 was replaced with alanine, showed a loss of reactivity with MAb TV20 (Fig. 4). This suggests that either D53 is an important residue involved directly in MAb TV20 binding, or that the alanine mutation at this position affected the conformation of the epitope in its interaction with MAb TV20.

**Figure 5:**
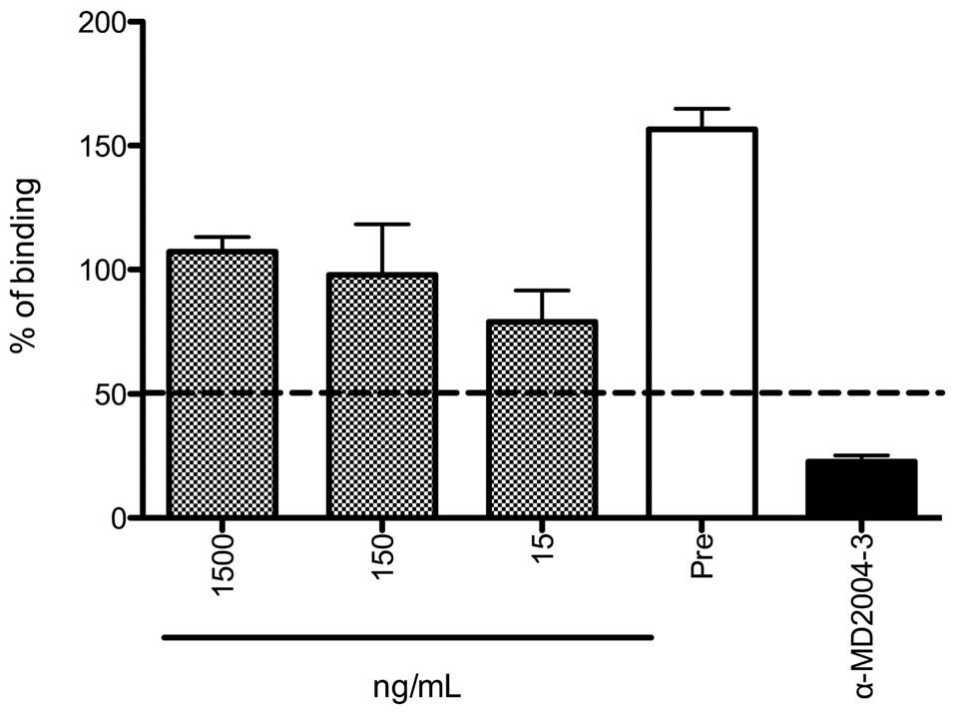
MAb TV20 fails to block the binding of GII.4 VLPs with histo-blood group antigens (HBGA). Percent binding of MD2004-3 (GII.4 strain) VLPs to B carbohydrate in the presence of different concentrations of MAb TV20 was calculated as described in *Materials and Methods*. Pre-immune sera and hyperimmune sera (α-MD2004-3) from immunized guinea pigs were used as negative and positive control, respectively. Error bars represent the S.E.M. Dashed line represents the 50% blocking cut-off value.

Norovirus VLPs interact with carbohydrates from the HBGA [2], [33], [35], and the blocking of norovirus HBGA binding sites by antibodies in the sera from human volunteers has been shown to correlate with protection from illness [36]. Because of this, assays that measure the blocking of VLP:HBGA interactions have been used as a surrogate for norovirus neutralization [26], [33], [36]. Although it would be expected that MAbs that map to the P domain will be involved in blocking the VLP:HBGA interaction, we recently showed that a GII.4 MAb that maps to the S domain could partially block VLP:HBGA interaction [14]. We tested the ability of MAb TV20 to block the interaction of GII.4 VLPs with HBGAs, but no blocking activity was detected at any of the concentrations tested (Fig. 5).

MAbs have proven useful as tools for study of viral immunity, pathology, epidemiology, and they may also have therapeutic applications [37]–[39]. The expression of norovirus VP1 protein has facilitated the development of MAbs, which have been important for studies of antigenic diversity as well as the development of diagnostic tests [11], [14], [17], [18], [20], [21], [23]–[25]. Several cross-reactive antibodies have been described and characterized for noroviruses. Most of them target the conserved S domain or the C-terminal region of the P1 domain [14], [17]–[20], [40]–[42], but none of them have been shown to target an epitope conserved across the entire *Norovirus* genus. Interestingly, a MAb developed against bovine strains (GIII), which maps to the S domain between residues 31–39, was shown to be reactive against VLPs from human GII.3 noroviruses, but not others [19]. Recently, Li *et al.* (2010) have identified a broadly cross-reactive MAb (N2C3) that binds to an epitope that spans residues 55–60 of the norovirus capsid protein. Although N2C3 can bind to multiple VLPs from GI, GII, GIII and GV strains, several strains (e.g. GI.3, GI.9, G.13, GII.5, GII.6, GII.14) have shown mutations that abolish the interaction with the MAb. In this study we developed and characterized a MAb that targets an epitope (corresponding to residues 52–56 of the NV VP1) mapping near to those described by Li *et al.* and Oliver *et al.*, and that is apparently highly conserved among all norovirus strains (Fig. 3a). After immunization with the capsid protein from a GII.3 strain, Yoda *et al.* noted that 6 out of 10 MAbs recovered bound to epitopes localized between residues 31–70 [40]. Together, these results suggest that the S domain is a highly immunogenic region of the VP1, and because it is highly conserved, is a good target for the development of cross-reactive antibodies.

Over the last two decades the awareness of norovirus as an important pathogen of gastroenteritis has risen, mostly due to the improvement of detection techniques [23]–[25], [43]. However, the lack of an *in vitro* culture system or a robust animal model, coupled with the great diversity associated with this virus, has hampered our knowledge of the immune response and pathology. Thus, this broadly cross-reactive MAb should prove useful in the diagnosis and study of noroviruses, and may lead to new insights in norovirus epidemiology, disease and vaccine development.

## References

[1] Patel MM, Widdowson MA, Glass RI, Akazawa K, Vinje J, et al (2008) Systematic literature review of role of noroviruses in sporadic gastroenteritis. Emerg Infect Dis 14: 1224–1231. 18680645 10.3201/eid1408.071114 PMC2600393

[2] Green KY (2007) Caliciviridae: The Noroviruses. In: Knipe DM, Howley PM, editors. Fields Virology. Philadelphia, Pa:Lippincott, Williams & Wilkins. pp. 949–979.

[3] Bertolotti-Ciarlet A, Crawford SE, Hutson AM, Estes MK (2003) The 3′ end of Norwalk virus mRNA contains determinants that regulate the expression and stability of the viral capsid protein VP1: a novel function for the VP2 protein. J Virol 77: 11603–11615. 14557646 10.1128/JVI.77.21.11603-11615.2003 PMC229252

[4] Jiang X, Wang M, Graham DY, Estes MK (1992) Expression, self-assembly, and antigenicity of the Norwalk virus capsid protein. J Virol 66: 6527–6532. 1328679 10.1128/jvi.66.11.6527-6532.1992 PMC240146

[5] Green KY, Lew JF, Jiang X, Kapikian AZ, Estes MK (1993) Comparison of the reactivities of baculovirus-expressed recombinant Norwalk virus capsid antigen with those of the native Norwalk virus antigen in serologic assays and some epidemiologic observations. J Clin Microbiol 31: 2185–2191. 8396590 10.1128/jcm.31.8.2185-2191.1993 PMC265719

[6] Atmar RL, Bernstein DI, Harro CD, Al-Ibrahim MS, Chen WH, et al (2011) Norovirus vaccine against experimental human Norwalk Virus illness. N Engl J Med 365: 2178–2187. 22150036 10.1056/NEJMoa1101245 PMC3761795

[7] Herbst-Kralovetz M, Mason HS, Chen Q (2010) Norwalk virus-like particles as vaccines. Expert Rev Vaccines 9: 299–307. 20218858 10.1586/erv.09.163 PMC2862602

[8] Tacket CO, Sztein MB, Losonsky GA, Wasserman SS, Estes MK (2003) Humoral, mucosal, and cellular immune responses to oral Norwalk virus-like particles in volunteers. Clin Immunol 108: 241–247. 14499247 10.1016/s1521-6616(03)00120-7

[9] Prasad BV, Hardy ME, Dokland T, Bella J, Rossmann MG, et al (1999) X-ray crystallographic structure of the Norwalk virus capsid. Science 286: 287–290. 10514371 10.1126/science.286.5438.287

[10] Debbink K, Donaldson EF, Lindesmith LC, Baric RS (2012) Genetic mapping of a highly variable norovirus GII.4 blockade epitope: potential role in escape from human herd immunity. J Virol 86: 1214–1226. 22090110 10.1128/JVI.06189-11 PMC3255819

[11] Lindesmith LC, Debbink K, Swanstrom J, Vinje J, Costantini V, et al (2012) Monoclonal antibody-based antigenic mapping of norovirus GII.4-2002. J Virol 86: 873–883. 22090098 10.1128/JVI.06200-11 PMC3255811

[12] Tan M, Fang P, Chachiyo T, Xia M, Huang P, et al (2008) Noroviral P particle: structure, function and applications in virus-host interaction. Virology 382: 115–123. 18926552 10.1016/j.virol.2008.08.047 PMC3508508

[13] Lochridge VP, Jutila KL, Graff JW, Hardy ME (2005) Epitopes in the P2 domain of norovirus VP1 recognized by monoclonal antibodies that block cell interactions. J Gen Virol 86: 2799–2806. 16186235 10.1099/vir.0.81134-0

[14] Parra GI, Abente EJ, Sandoval-Jaime C, Sosnovtsev SV, Bok K, et al (2012) Multiple Antigenic Sites are Involved in Blocking the Interaction of GII.4 Norovirus Capsid with ABH Histo-Blood Group Antigens. J Virol 86: 7414–7426. 22532688 10.1128/JVI.06729-11 PMC3416322

[15] Mesquita JR, Barclay L, Nascimento MS, Vinje J (2010) Novel norovirus in dogs with diarrhea. Emerg Infect Dis 16: 980–982. 20507751 10.3201/eid1606.091861 PMC3086253

[16] Duffy S, Shackelton LA, Holmes EC (2008) Rates of evolutionary change in viruses: patterns and determinants. Nat Rev Genet 9: 267–276. 18319742 10.1038/nrg2323

[17] Parker TD, Kitamoto N, Tanaka T, Hutson AM, Estes MK (2005) Identification of Genogroup I and Genogroup II broadly reactive epitopes on the norovirus capsid. J Virol 79: 7402–7409. 15919896 10.1128/JVI.79.12.7402-7409.2005 PMC1143648

[18] Batten CA, Clarke IN, Kempster SL, Oliver SL, Bridger JC, et al (2006) Characterization of a cross-reactive linear epitope in human genogroup I and bovine genogroup III norovirus capsid proteins. Virology 356: 179–187. 16934306 10.1016/j.virol.2006.07.034

[19] Oliver SL, Batten CA, Deng Y, Elschner M, Otto P, et al (2006) Genotype 1 and genotype 2 bovine noroviruses are antigenically distinct but share a cross-reactive epitope with human noroviruses. J Clin Microbiol 44: 992–998. 16517888 10.1128/JCM.44.3.992-998.2006 PMC1393167

[20] Shiota T, Okame M, Takanashi S, Khamrin P, Takagi M, et al (2007) Characterization of a broadly reactive monoclonal antibody against norovirus genogroups I and II: recognition of a novel conformational epitope. J Virol 81: 12298–12306. 17855545 10.1128/JVI.00891-07 PMC2168978

[21] Li X, Zhou R, Tian X, Li H, Zhou Z (2010) Characterization of a cross-reactive monoclonal antibody against Norovirus genogroups I, II, III and V. Virus Res. 151: 142–147. 10.1016/j.virusres.2010.04.005 20417671

[22] Parra GI, Bok K, Taylor R, Haynes JR, Sosnovtsev SV, et al (2012) Immunogenicity and specificity of norovirus Consensus GII.4 virus-like particles in monovalent and bivalent vaccine formulations. Vaccine 30: 3580–3586. 22469864 10.1016/j.vaccine.2012.03.050 PMC3359014

[23] Geginat G, Kaiser D, Schrempf S (2011) Evaluation of third-generation ELISA and a rapid immunochromatographic assay for the detection of norovirus infection in fecal samples from inpatients of a German tertiary care hospital. Eur J Clin Microbiol Infect Dis 31: 733–737. 21809086 10.1007/s10096-011-1366-z

[24] Kirby A, Gurgel RQ, Dove W, Vieira SC, Cunliffe NA, et al (2010) An evaluation of the RIDASCREEN and IDEIA enzyme immunoassays and the RIDAQUICK immunochromatographic test for the detection of norovirus in faecal specimens. J Clin Virol 49: 254–257. 20864394 10.1016/j.jcv.2010.08.004

[25] de Bruin E, Duizer E, Vennema H, Koopmans MP (2006) Diagnosis of Norovirus outbreaks by commercial ELISA or RT-PCR. J Virol Methods 137: 259–264. 16901556 10.1016/j.jviromet.2006.06.024

[26] Bok K, Parra GI, Mitra T, Abente E, Shaver CK, et al (2011) Chimpanzees as an animal model for human norovirus infection and vaccine development. Proc Natl Acad Sci U S A 108: 325–330. 21173246 10.1073/pnas.1014577107 PMC3017165

[27] Lew JF, Kapikian AZ, Jiang X, Estes MK, Green KY (1994) Molecular characterization and expression of the capsid protein of a Norwalk-like virus recovered from a Desert Shield troop with gastroenteritis. Virology 200: 319–325. 8128635 10.1006/viro.1994.1194

[28] Leite JP, Ando T, Noel JS, Jiang B, Humphrey CD, et al (1996) Characterization of Toronto virus capsid protein expressed in baculovirus. Arch Virol 141: 865–875. 8678832 10.1007/BF01718161

[29] Holzinger A, Phillips KS, Weaver TE (1996) Single-step purification/solubilization of recombinant proteins: application to surfactant protein B. Biotechniques 20: 804–806, 808. 10.2144/96205bm16 8723923

[30] Kohler G, Milstein C (1975) Continuous cultures of fused cells secreting antibody of predefined specificity. Nature 256: 495–497. 1172191 10.1038/256495a0

[31] Wyatt LS, Moss B, Rozenblatt S (1995) Replication-deficient vaccinia virus encoding bacteriophage T7 RNA polymerase for transient gene expression in mammalian cells. Virology 210: 202–205. 7793072 10.1006/viro.1995.1332

[32] LoBue AD, Lindesmith L, Yount B, Harrington PR, Thompson JM, et al (2006) Multivalent norovirus vaccines induce strong mucosal and systemic blocking antibodies against multiple strains. Vaccine 24: 5220–5234. 16650512 10.1016/j.vaccine.2006.03.080

[33] Harrington PR, Lindesmith L, Yount B, Moe CL, Baric RS (2002) Binding of Norwalk virus-like particles to ABH histo-blood group antigens is blocked by antisera from infected human volunteers or experimentally vaccinated mice. J Virol 76: 12335–12343. 12414974 10.1128/JVI.76.23.12335-12343.2002 PMC136916

[34] Pettersen EF, Goddard TD, Huang CC, Couch GS, Greenblatt DM, et al (2004) UCSF Chimera--a visualization system for exploratory research and analysis. J Comput Chem 25: 1605–1612. 15264254 10.1002/jcc.20084

[35] Tan M, Jiang × (2010) Norovirus gastroenteritis, carbohydrate receptors, and animal models. PLoS Pathog 6: e1000983. 20865168 10.1371/journal.ppat.1000983 PMC2928792

[36] Reeck A, Kavanagh O, Estes MK, Opekun AR, Gilger MA, et al (2010) Serological correlate of protection against norovirus-induced gastroenteritis. J Infect Dis 202: 1212–1218. 20815703 10.1086/656364 PMC2945238

[37] Kwong PD, Wilson IA (2009) HIV-1 and influenza antibodies: seeing antigens in new ways. Nat Immunol 10: 573–578. 19448659 10.1038/ni.1746 PMC2796958

[38] Donaldson EF, Lindesmith LC, Lobue AD, Baric RS (2010) Viral shape-shifting: norovirus evasion of the human immune system. Nat Rev Microbiol 8: 231–241. 20125087 10.1038/nrmicro2296 PMC7097584

[39] Balazs AB, Chen J, Hong CM, Rao DS, Yang L, et al (2011) Antibody-based protection against HIV infection by vectored immunoprophylaxis. Nature 481: 81–84. 22139420 10.1038/nature10660 PMC3253190

[40] Yoda T, Terano Y, Suzuki Y, Yamazaki K, Oishi I, et al (2000) Characterization of monoclonal antibodies generated against Norwalk virus GII capsid protein expressed in Escherichia coli. Microbiol Immunol 44: 905–914. 11145271 10.1111/j.1348-0421.2000.tb02582.x

[41] Li X, Zhou R, Wang Y, Sheng H, Tian X, et al (2009) Identification and characterization of a native epitope common to norovirus strains GII/4, GII/7 and GII/8. Virus Res 140: 188–193. 19121346 10.1016/j.virusres.2008.12.004

[42] Almanza H, Cubillos C, Angulo I, Mateos F, Caston JR, et al (2008) Self-assembly of the recombinant capsid protein of a swine norovirus into virus-like particles and evaluation of monoclonal antibodies cross-reactive with a human strain from genogroup II. J Clin Microbiol 46: 3971–3979. 18842943 10.1128/JCM.01204-08 PMC2593275

[43] Atmar RL, Estes MK (2006) The epidemiologic and clinical importance of norovirus infection. Gastroenterol Clin North Am 35: :275–290, viii. 10.1016/j.gtc.2006.03.001 16880066

